# Mechanical and Microstructural Characteristics of Calcium Sulfoaluminate Cement Exposed to Early-Age Carbonation Curing

**DOI:** 10.3390/ma14133515

**Published:** 2021-06-24

**Authors:** Weikang Wang, Xuanchun Wei, Xinhua Cai, Hongyang Deng, Bokang Li

**Affiliations:** State Key Laboratory of Water Resources and Hydropower Engineering Science, Wuhan University, Wuhan 430072, China; wkwang@whu.edu.cn (W.W.); xuanchun.wei@foxmail.com (X.W.); denghy@whu.edu.cn (H.D.); libokang@whu.edu.cn (B.L.)

**Keywords:** calcium sulfoaluminate (CSA) cement, carbonation curing, pore structure, mechanical property, microstructure

## Abstract

The early-age carbonation curing technique is an effective way to improve the performance of cement-based materials and reduce their carbon footprint. This work investigates the early mechanical properties and microstructure of calcium sulfoaluminate (CSA) cement specimens under early-age carbonation curing, considering five factors: briquetting pressure, water–binder (w/b) ratio, starting point of carbonation curing, carbonation curing time, and carbonation curing pressure. The carbonization process and performance enhancement mechanism of CSA cement are analyzed by mercury intrusion porosimetry (MIP), thermogravimetry and derivative thermogravimetry (TG-DTG) analysis, X-ray diffraction (XRD), and scanning electron microscope (SEM). The results show that early-age carbonation curing can accelerate the hardening speed of CSA cement paste, reduce the cumulative porosity of the cement paste, refine the pore diameter distribution, and make the pore diameter distribution more uniform, thus greatly improving the early compressive strength of the paste. The most favorable w/b ratio for the carbonization reaction of CSA cement paste is between 0.15 and 0.2; the most suitable carbonation curing starting time point is 4 h after initial hydration; the carbonation curing pressure should be between 3 and 4 bar; and the most appropriate time for carbonation curing is between 6 and 12 h.

## 1. Introduction

Since the beginning of the 21st century, infrastructure construction projects in various fields have been rapidly developing and improving in China. Across the various types of construction projects, cement has always been one of the most widely used construction materials, so China’s annual production and use of cement has always been at the forefront of the world in recent times [1]. However, the production of cement is also accompanied by high energy consumption, high emissions, and serious pollution, and the production of 1 ton of cement in China produces more than 900 tons of CO_2_, which is a great challenge for the country’s low-carbon, green sustainable development strategy [2,3]. In 2017, the reported global CO_2_ emissions reached 36 Gt, an increase of almost 50% compared with CO_2_ emissions twenty years previously [4]. The cement industry produces a mass of CO_2_ gas that accounts for approximately 5% of the global CO_2_ emissions, which has become a major environmental problem [5]. In order to address the issue of global warming and reduce the carbon footprint of the building materials industry, experts and scholars proposed the idea of carbonation curing technology as early as the 1970s. With the deteriorating global environment, this technology has attracted increasing attention. It has been estimated that approximately 1.5 million metric tons of CO_2_ can be sequestered annually by carbonation curing of concrete masonry units, which will lead to a reduction of nearly 3% in carbon emissions [6]. Carbonization of cement-based materials refers to the process by which external CO_2_ penetrates into the material and reacts with hydration products in the cement-based material with the participation of water, reducing the alkalinity of the material, also known as the neutralization of cement-based materials [7,8,9]. Some experts and scholars have found that the introduction of a high concentration of CO_2_ in the early hydration of cement-based materials can greatly improve the hardening rate of the materials, effectively improve the mechanical properties, and improve the resistance of materials to wetting/drying and freeze/thaw cycles, sulfate attack, and acid erosion. Therefore, early-age carbonation curing technology is an effective way to reduce the carbon footprint and improve all aspects of material performance [10,11].

In natural air, the carbon dioxide in the air and the saturated calcium hydroxide solution in concrete pores undergo a natural carbonation reaction, the pH value of the pore solution in the concrete decreases, and when the carbonation depth exceeds the protective layer, the concrete loses its protective effect on the reinforcement and the steel begins to corrode under the conditions of coexistence of water and air. Early carbonation is different from weathering carbonation [12]. The CO_2_ volume is approximately 0.03% of the air, and thus the natural carbonation reaction is quite slow, and the CO_2_ diffusion coefficient of concrete is approximately 10^−8^ cm^2^s^−1^ [5]. This passive carbonation process is slow and detrimental to mature cement-based materials [4]. However, early-age carbonation curing can sequester CO_2_ in just a few hours to a few days, and it has already been shown to improve strength and durability performance by altering the chemical composition and microstructure of concrete products [4,12,13].

In early carbonization curing, a suitable water–cement ratio should be selected to reduce adverse effects. The CO_2_ is dissolved in the aqueous phase in the pores and transformed into carbonic acid (H_2_CO_3_), which is dissociated in ions (HCO_3_^−^ and CO_3_^2−^). Additionally, the calcium hydroxide (Ca(OH)_2_) is dissolved in Ca^2+^ and OH ions, resulting in the precipitation of calcium carbonate (CaCO_3_). Therefore, a minimum content of water is essential for the ionization of compounds to promote the reaction of carbonation, but a large amount of water (i.e., saturated pores) limits the reaction due to the blockage of the pores [14]. In laboratory mechanistic studies, w/b ratios below 0.2 are often used, whereas a water removal process prior to the early-age carbonation is needed for mixes with higher w/b ratios. The purpose of using such a low w/b ratio is to exclude the effect of excessive water on CO_2_ diffusion and to minimize experimental variation by averting the water removal process [13].

**Figure 1:**
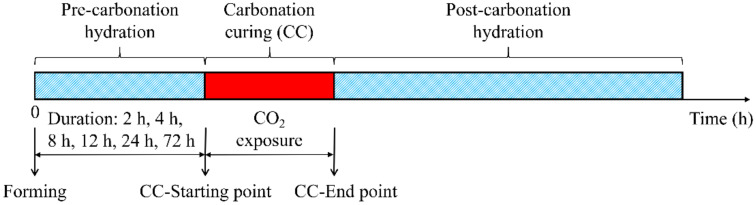
Early-age carbonation curing regime of specimen.

As shown in Figure 1, each early-age carbonation scenario contains three steps: pre-carbonation hydration, CO_2_ exposure (carbonation curing), and post-carbonation hydration after the specimen is formed [13]. Unlike weathering carbonization, hydrated cement paste develops strength faster and improves long-term durability during early carbonation curing, usually within 24 h [12,13]. The compressive strength of CO_2_-cured cement paste increased with curing time within 24 h, and the increment was especially striking in the first 2 h [15]. Carbonation curing leads to a significant improvement in the early strength of cement mortars, but with the extension of the curing age, this positive effect gradually weakens, and excessive carbonation will lead to decalcification of C-S-H and decrease in strength [16]. Therefore, it is very important to choose the proper carbonation curing time.

Chen and Gao [6] summarized a series of physicochemical reactions that occur during early accelerated carbonization: (i) phase transition and mass transfer of CO_2_; (ii) dissolution of solid Ca(OH)_2_ and mass transfer of dissolved Ca(OH)_2_; (iii) hydration of cement compounds; (iv) carbonation of unhydrated cement compounds and hydration products. The mechanism and kinetics of these reactants of carbonation are different. The carbonation mechanism of CH is a direct reaction, but the carbonation mechanism of C-S-H is more complicated [16] (see Equations (1) and (2)). C_3_S and its hydration product Ca(OH)_2_ are the key reactants for early-age carbonation. The carbonation of C_3_S and Ca(OH)_2_ is prior to that of C_2_S and C–S–H. While the carbonation of C_2_S and C-S-H has a small level of influence on the total carbonation, all the carbonation reactants involved compete for capturing carbon dioxide [6]. Despite a similar reaction mechanism, the hydration reaction of C_2_S results in lower production of calcium hydroxide (CH) and heat as compared to C_3_S (see Equations (3) and (4)). Carbonation of fresh state C_2_S and C_3_S results in an accelerated curing process and is governed by the reactions (see Equations (5) and (6)) [17].

(1) $$\mathrm{Ca}{\left(\mathrm{OH}\right)}_{2}+{\mathrm{CO}}_{2}\to {\mathrm{CaCO}}_{3}+H_{2}O$$

(2) $$C-S-H+{\mathrm{CO}}_{2}\to {\mathrm{CaCO}}_{3}+{\mathrm{SiO}}_{2}+H_{2}O$$

(3) $$C_{3}S+\left(3+m-n\right)H_{2}O\to C_{n}{\mathrm{SH}}_{m}+\left(3-n\right)\mathrm{CH}$$

(4) $$C_{2}S+\left(1.5+n\right)H_{2}O\to C_{1.5+m}{\mathrm{SH}}_{1+m+n}+\left(0.5-m\right)\mathrm{CH}$$

(5) $$C_{3}S+\left(3-x\right){\mathrm{CO}}_{2}+{\mathrm{yH}}_{2}O\to C_{x}{\mathrm{SH}}_{y}+\left(3-x\right){\mathrm{CaCO}}_{3}$$

(6) $$C_{2}S+\left(2-x\right){\mathrm{CO}}_{2}+{\mathrm{yH}}_{2}O\to C_{x}{\mathrm{SH}}_{y}+\left(2-x\right){\mathrm{CaCO}}_{3}$$

Small-scale non-reinforced concrete, such as masonry blocks and pavers, appears to be the most suitable candidate for carbonation curing [13]. Recent studies have shown that early carbonization curing can be effective in improving the physical and mechanical properties of ground granulated blast-furnace slag (GGBFS) mineral admixtures [18], recycled aggregate (RA) and prestressed concrete [5], Portland limestone cement (PLC) [13], vegetable fiber-reinforced cement composite [14], belite-rich cement [17], ordinary Portland cement (OPC) [12,13,17], concrete blocks incorporating drinking water treatment sludge [4], Portland cement and lime-pozolan composites [19], and cement mortars containing cement kiln dust [7], thus meeting the application requirements. However, there are still relatively few studies on the performance of early carbonation curing of calcium sulfoaluminate cement.

Calcium sulfoaluminate (CSA) cement is a hydraulic binding material with high early strength. It is made by calcining appropriate raw materials to obtain clinker with anhydrous calcium sulfoaluminate (3CaO·3Al_2_O_3_·CaSO_4_, abbreviated as $C_{4}A_{3}\bar{S}$) and dicalcium silicate (Ca_2_SiO_4_, abbreviated as C_2_S) as the main mineral components, and then adding an appropriate amount of gypsum to grind [20]. The hydration process of sulfoaluminate cement is shown in Equations (7)–(10), and the hydration products are mainly ettringite (AFt, $C_{6}A{\bar{S}}_{3}H_{32}$), monosulfate (AFm, $C_{4}A\bar{S}H_{12}$), alumina hydroxide gel (AH_3_), C-S-H gel, and a small amount of calcium hydroxide (CH) [21,22,23]. CSA cement has the characteristics of fast setting speed, high early strength, good impermeability, micro expansion, and low alkalinity. It is often used in emergency repair projects, such as joints, leak plugging, bolt-shotcrete, and emergency repair of aircraft runways, etc. However, in some special cases, such as winter construction or special materials in some engineering parts, the requirements for the hydration and hardening speed and early strength of CSA cement are higher [24,25]. Deng studied the influence of different anions of three soluble lithium compounds (Li_2_CO_3_, Li_2_SO_4_, and LiCl) on the hydration properties of CSA cement and revealed the mechanism of the accelerating effect of lithium compounds, providing support for the application of different lithium compounds in CSA cement [26].

(7) $$C_{4}A_{3}\bar{S}+2C\bar{S}H_{2}+36H\to \mathrm{AFt}+2{\mathrm{AH}}_{3}\left(\mathrm{gel}\right)$$

(8) $$C_{4}A_{3}\bar{S}+18H\to 2{\mathrm{AH}}_{3}+\mathrm{AFm}$$

(9) $$C_{2}S+\mathrm{nH}\to C-S-H+\mathrm{CH}$$

(10) $${\mathrm{AH}}_{3}\left(\mathrm{gel}\right)+3\mathrm{CH}+3C\bar{S}H_{2}+20H\to \mathrm{AFt}$$

In this paper, the early-age carbonation curing of CSA cement is carried out to improve the hardening rate and enhance the early mechanical properties of specimens. This work studies the influence of five different factors on the early-age carbonation performance of the material. Through mercury intrusion porosimetry (MIP) and other test methods to analyze the carbonation process of CSA cement and the mechanism of performance enhancement, this study provides a reference for accelerating the hardening speed of CSA cement and reducing the carbon footprint of materials in projects.

## 2. Materials and Methods

### 2.1. Materials

The chemical composition of the 42.5 grade rapid hardening composite CSA cement produced by Polar Bear Building Materials Company in Tangshan, Hebei, China, was as shown in Table 1 [26], with Al_2_O_3_ content of 18.77%, CaO content as high as 47.56%, and high content of SO_3_ and CO_2_, due to the decomposition of calcium sulfate (CaSO_4_) and calcium sulfoaluminate ($C_{4}A_{3}\bar{S}$) during the testing process. The physical properties and quality tests of CSA cement are shown in Table 2. The measured specific surface area reached 446 m^2^/kg, which was greater than the standard requirement of 350 m^2^/kg. The initial and final setting times were also within the required range, and the flexural strength and compressive strength at 1 d reached 7.4 MPa and 44.5 MPa, respectively. Therefore, all the quality indexes are in accordance with the Chinese Standard GB/T 20472-2006 [27].

In this study, high-purity CO_2_ gas produced by Wuhan Minghui Gas Co., Ltd. with a purity of 99% was used in order to accelerate the carbonization process of CSA cement test blocks and shorten the test cycle. The water used for forming the specimens was deionized by a stainless steel automatic distilled water machine (product model ID: YN-ZD-Z-20, Shanghai Lichen Bangxi Instrument Co., Ltd., Shanghai, China) to avoid the effects of some of the ions contained in the tap water.

### 2.2. Test Methods and Sample Preparation

**Figure 2:**
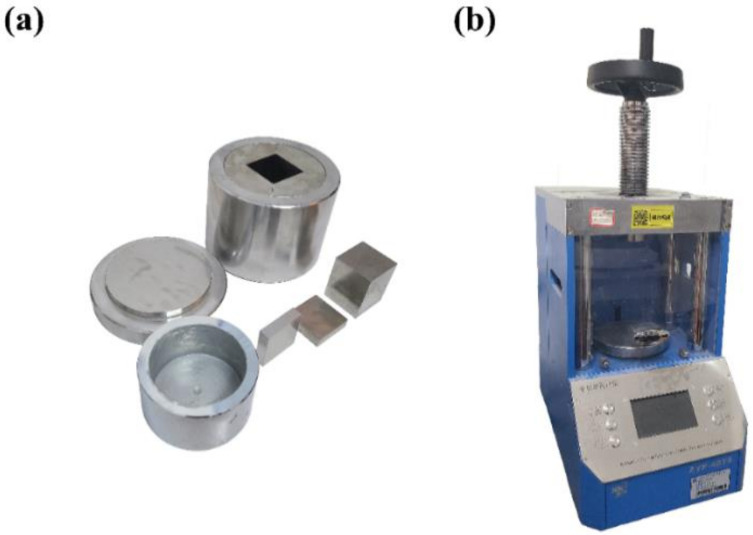
Sampling equipment: (**a**) cylindrical opening valve mold, (**b**) automatic powder tablet press.

**Figure 3:**
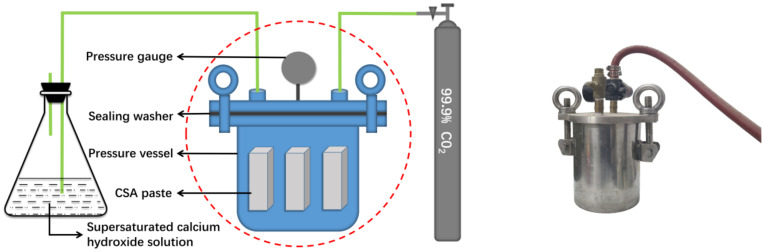
Carbonation curing device.

At a high water–binder ratio, too much pore fluid in the paste affects the effect of early-age carbonation curing, i.e., it is difficult for CO_2_ to enter the interior of the CSA cement block at a high water–binder ratio. In this study, in order to ensure the effect of carbonation curing, the w/b ratio of the sample is not more than 0.22, and the sample is formed by mechanical pressure. The CSA cement was mixed with water according to a certain w/b ratio, poured into the cylindrical split mold, and then shaped under different pressures applied by the fully automatic powder compactor produced by Shanghai Xinno Instrument Co. The cylindrical split mold used in this test is shown in Figure 2a. The overall shape of the mold is cylindrical, with a square 40 mm × 40 mm × (0~70) mm inner cavity in the center. The automatic powder compactor is shown in Figure 2b; the product model is ZYP-40TS, and the pressure range is 0–40 tons. The mixed cement paste was poured into the automatic power compactor and pressed into a cube specimen with the size of 40 mm × 40 mm × 40 mm. The carbonation curing device used in the test is shown in Figure 3, which consists of a pressure vessel, piping, pressure gauges, safety valves, and CO_2_ cylinders. The test procedure was as follows: first, put the formed test block into the pressure vessel, then tighten the lid of the vessel using the screw above the vessel to prevent air leakage, open the safety valve above the pressure vessel, and then turn on the switch of the CO_2_ cylinder to let the CO_2_ gas pass. Close the safety valve after the gas has entered the pressure vessel for about 1 min [28]. The purpose of this operation is to evacuate the air from the pressure vessel and to ensure that only CO_2_ gas is present in the vessel during the test. Finally, slowly increase the air intake until the corresponding curing pressure is displayed on the pressure gauge, and then cure the test block to the corresponding age. The testing methods used in the test are cube compressive strength test, mercury intrusion porosimetry (MIP) test, thermogravimetric (TG) analysis, X-ray diffraction (XRD) test, and scanning electron microscope (SEM). The mercury intrusion test uses the PoreMaster/PoreMaster GT type mercury intrusion porosimeter produced by Quantachrome, Boynton Beach, FL, USA. The voltage of the instrument is 220–240 V, the frequency is 50 Hz or 60 Hz, and the maximum power is 1000 VA.

## 3. Results and Discussion

### 3.1. Briquetting Pressure

**Figure 4:**
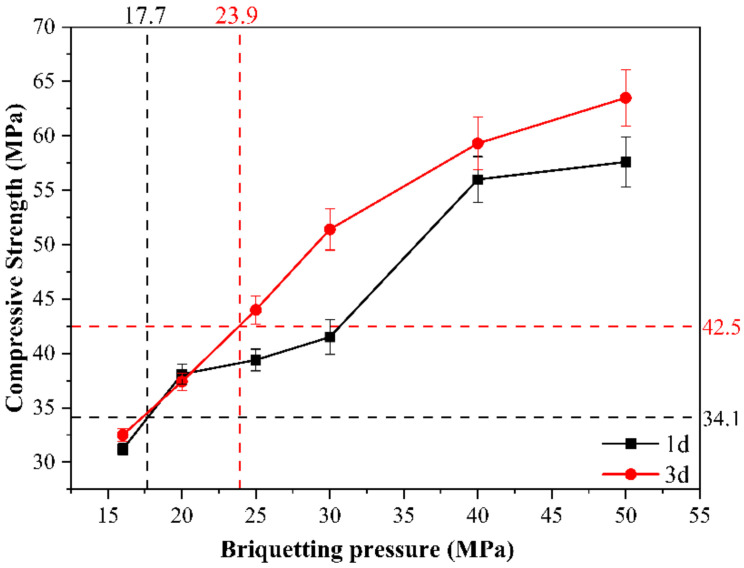
Influence of briquetting pressure on compressive strength of CSA cement pastes.

Figure 4 presents the compressive strengths of 0.15 w/b ratio and different pressures of the test blocks of CSA cement paste formed to 1 d and 3 d curing, respectively. As can be seen from Figure 4, when the briquetting pressure is increased, the compressive strength of the test block increases. First, the test needs to set a pressure value which has little influence on the strength development. When the briquetting pressure is too small (<15 MPa), the specimen cannot be formed. When it is too high (>25 MPa), the compressive strength caused by the pressure is far greater than that caused by the hydration of cement itself. When the briquetting pressure of the automatic powder tablet press reaches 50 MPa, the 3 days compressive strength of the test block can exceed 62.5 MPa. However, the compressive strength and the briquetting pressure are not linearly related. When the briquetting pressure increases to a certain extent, the growth rate of the compressive strength gradually slows down. This corresponds to the objective reality that the compressive strength of a material cannot grow indefinitely, even if the briquetting pressure is increased, because the strength of the material itself is limited. From Figure 4, it is obvious that when the briquetting pressure is less than 20 MPa, the compressive strength of the test block at the age of 3 days does not differ much from the strength at 1 day. When the briquetting pressure exceeds 20 MPa, the gap between the strength at 1 d and 3 days gradually increases. The two methods of specimen preparation were compared by mechanical pressure forming and traditional direct water mixing. When using traditional sample preparation methods without briquetting pressure, the one-day compressive strength of CSA cement paste with the common water–binder ratio of 0.35 is 34.1 MPa, and the three-day compressive strength is 42.5 MPa under standard curing conditions, which is similar to the compressive strength of specimens with briquetting pressures between 17.7 and 23.9 MPa in Figure 4. Therefore, in order to facilitate comparison of test data results, the briquetting pressure of 20 MPa is used in the subsequent tests in this section.

### 3.2. Water-to-Binder Ratio

In this test, the water–binder ratios used are 0.05, 0.10, 0.15, 0.20, and 0.22. According to the test results in the above Section 3.1, the briquetting pressure of the sample is kept at 20 MPa in the subsequent test, and the performance changes of CSA cement pastes with different w/b ratios after carbonation curing are studied. The carbonation curing starting point is 4 h after initial hydration, and the curing pressure is 1 bar.

**Figure 5:**
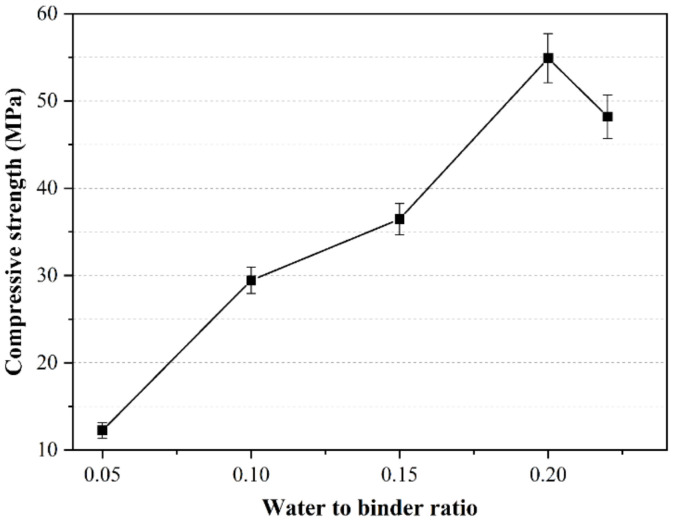
Influence of water-to-binder ratio on compressive strength of CSA cement pastes.

The compressive strength test results are shown in Figure 5. When the w/b ratio is 0.05, the compressive strength of the test block after carbonation curing is very low, at only about 12 MPa. When the w/b ratio is gradually increased to 0.2, the compressive strength of the test block gradually rises. After 2 h of carbonation curing, the compressive strength of the test block with a w/b ratio of 0.2 is nearly 55 MPa, which is much higher than the strength of the 3 days test block under standard curing conditions. This shows that carbonation curing can greatly promote the hardening rate of the paste and improve the early compressive strength. When the w/b ratio continues to increase to 0.22, the compressive strength decreases instead. This is due to the fact that there will be more water in the pores of the cement paste as we continue to increase the water-to-binder ratio. In this case, the carbonation curing will reduce the diffusion rate of CO_2_ into the paste through the pores in the paste, and the carbonization reaction cannot fully occur inside the test block, resulting in a decrease in compressive strength. Therefore, the w/b ratio that is most conducive to the carbonization reaction of the CSA cement paste and the improvement of the early compressive strength of the paste is between 0.15 and 0.2.

**Figure 6:**
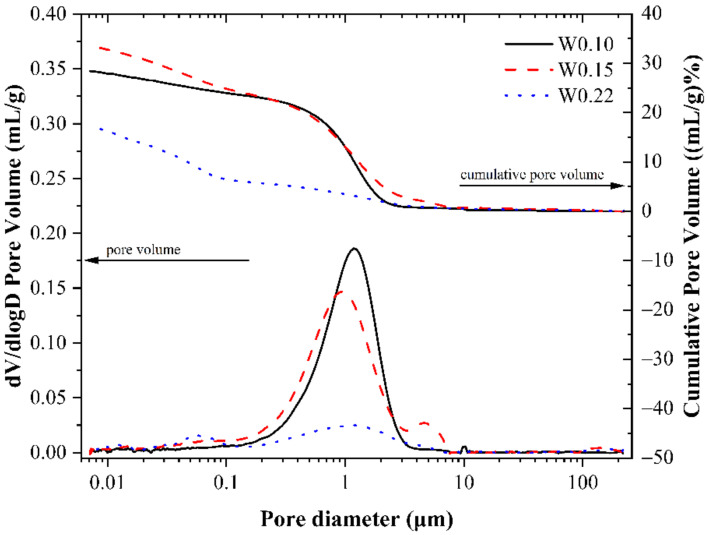
Pore volume distribution of CSA cement pastes at different water-to-binder ratios.

The results of the mercury intrusion test are as follows. Figure 6 illustrates the cumulative porosity versus pore diameter distribution curve and the differential curve of the relationship between the cumulative porosity and the pore diameter distribution, and the letter “W” is used to indicate the w/b ratio. From Figure 6, it can be seen that the cumulative porosity of the sample with a w/b ratio of 0.15 is the largest, reaching 33.49%. The sample with a w/b ratio of 0.22 has the lowest cumulative porosity, at only 17.30%, and the sample with a w/b ratio of 0.1 has a cumulative porosity of 28.35%. It can be seen that the highest peak value of the differential curve of the W0.15 sample appears at 0.9315 μm, the highest peak value of the differential curve of the W0.1 sample appears at 1.187 μm, and the highest peak value of the differential curve of the W0.22 sample appears at 1.257 μm. Although the cumulative porosity of the sample with a w/b ratio of 0.15 is the highest, the diameter of the pore with the highest proportion is the smallest. Although the cumulative porosity of the sample W0.22 with the highest w/b ratio is the smallest, the diameter of the pore with the highest proportion is the largest. This is because the sample with a w/b ratio of 0.15 has the highest degree of carbonation during the carbonation curing process. CO_2_ reacts with the mineral phase and hydration products in the cement paste, and the carbonized reaction products such as calcium carbonate (CaCO_3_) formed by the reaction can be filled in the pores of the cement paste, thereby refining the pores. Therefore, the compressive strength of the W0.15 sample is the highest. The W0.15 sample has the highest cumulative porosity, because under a certain carbonation curing pressure, CO_2_ will gradually diffuse into the test block, and the porosity of the sample will increase accordingly during the diffusion process. The W0.1 sample is relatively loose, and there is very little water in the pores. CO_2_ can easily enter the inside of the test block, so the porosity will not increase. The porosity of the W0.22 sample is relatively low. Due to the high degree of hydration, a large amount of hydration product is generated and accumulated in the pores. In addition, it can be seen from Figure 6 that the differential curves corresponding to the pore diameter distributions of the three w/b ratios are obviously in a normal distribution trend, and most of them are concentrated in the range of 0.1298–3.2178 μm. The pore diameter distribution in the cement paste is relatively uniform.

**Figure 7:**
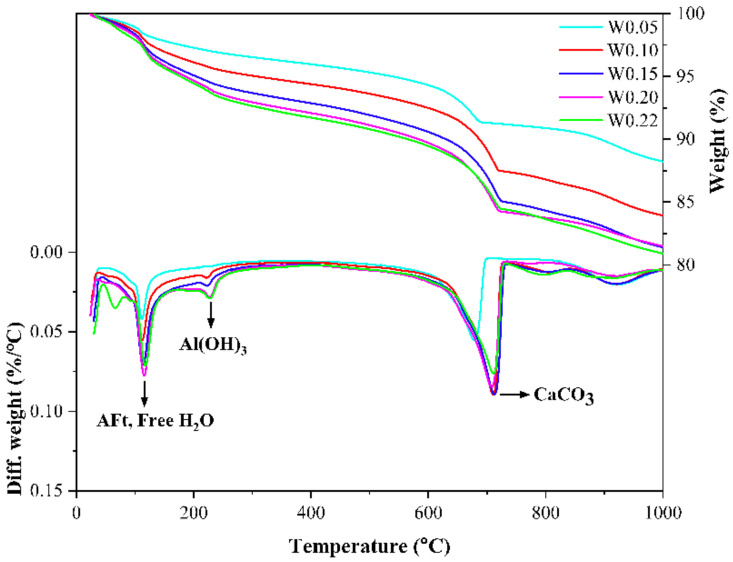
TG-DTG curves of CSA cement pastes corresponding to different water-to-binder ratios.

Figure 7 shows the TG-DTG curves of CSA cement pastes with different w/b ratios. It can be found that the height of the weight loss peak on the DTG curve increases with the increase in the w/b ratio in the temperature ranges of 80–150 °C and 200–250 °C, which correspond to the thermal decomposition of AFt and alumina hydroxide, respectively. In the temperature range of 600–800 °C corresponding to the thermal decomposition of CaCO_3_, the weight loss of W0.15 and W0.2 is the highest, indicating that the amount of CaCO_3_ in these two groups of samples after carbonation curing is the largest, which further illustrates that the most suitable w/b ratio for carbonation curing is between 0.15 and 0.2. The results shown by the TG-DTG curve are consistent with the conclusions in the compressive strength test [29].

**Figure 8:**
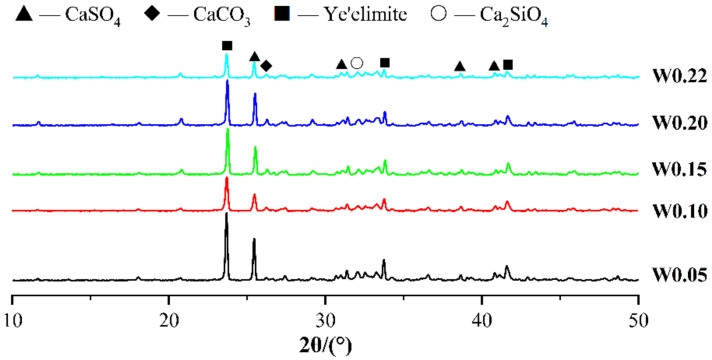
XRD patterns of CSA cement pastes corresponding to different water-to-binder ratios.

**Figure 9:**
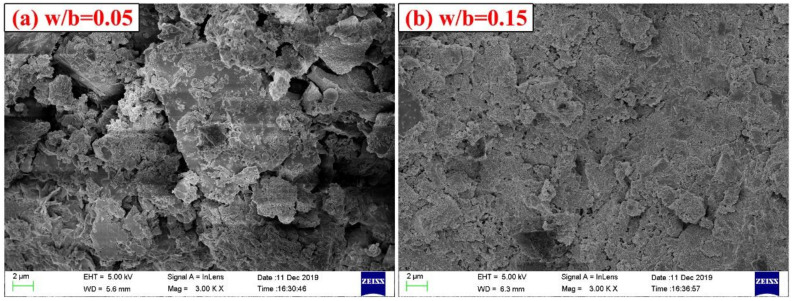
SEM images of CSA cement pastes corresponding to different water-to-binder ratios: (**a**) w/b = 0.05; (**b**) w/b = 0.15.

Figure 8 shows the XRD patterns of CSA cement pastes with different w/b ratios. As the w/b ratio increases, the diffraction peaks of ye’elimite gradually decrease, which means the degree of hydration increases. The W0.22 sample had the highest intensity of the diffraction peak for calcium sulfate, indicating that the W0.22 sample was more hydrated and more calcium sulfate was involved in the reaction. In the XRD patterns, the diffraction intensity of CaCO_3_ is very low, and there is no obvious diffraction peak in the diffraction angle range of 25–30°, which is inconsistent with the result of TG comprehensive thermal analysis. This may be due to the fact that the carbonization reaction of the CSA cement paste occurs in a very short time under carbonation curing at a certain pressure, and the formed CaCO_3_ has poor crystallization and irregular crystal structure. Therefore, even if the paste carbonization reaction generates a large amount of CaCO_3_, it cannot be detected in the XRD pattern. There are some little diffraction peaks at the position of 26–30° on the XRD pattern, indicating that the crystal structure of CaCO_3_ generated by the paste carbonization reaction is calcite and aragonite. In addition, there is no obvious diffraction peak of AFt on the pattern, indicating that AFt has been transformed under carbonation curing. Figure 9a,b shows the SEM images of W0.05 and W0.15 samples, respectively. It can be found that under SEM magnification of 3000 times, the structure of the W0.05 sample is very loose and a large amount of cement mineral has not been hydrated, whereas the W0.15 sample was relatively much denser in structure, indicating a much higher degree of hydration than the W0.05 sample. The crystal shape of CaCO_3_ seen under SEM is not a regular cubic shape, but is in block, granular, or columnar shape, which also explains why there is no obvious diffraction peak of CaCO_3_ in the XRD pattern.

### 3.3. Carbonation Starting Time Point

**Figure 10:**
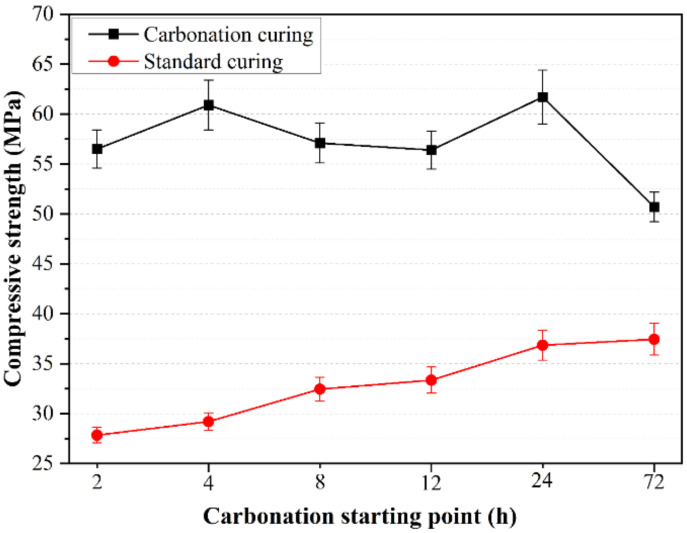
Influence of carbonation starting point on compressive strength of CSA cement pastes.

The control variable method is also used in this section. The briquetting pressure of the sample is 20 MPa, and the w/b ratio is 0.15. Under six different starting points of carbonation (2 h, 4 h, 8 h, 12 h, 1 day, and 3 days), the changes in various properties of samples after 6 h of carbonation curing at 4 bar curing pressure were studied. The compressive strength test results are shown in Figure 10. Compared with the standard curing samples at the same time point, the compressive strength of the samples after carbonation curing is much higher. The compressive strengths after carbonation curing are all above 50 MPa, while the strength of the specimens only reaches 37 MPa after 3 d of the standard curing, which shows that early-age carbonation curing can greatly improve the early mechanical properties of CSA cement paste. Comparing the strength of carbonized and non-carbonized samples, it is found that the difference in compressive strength of the samples corresponding to carbonation curing and standard curing reaches the maximum when the carbonation curing starting point is 4 h. As the carbonation curing starting point goes on, the difference in compressive strength becomes increasingly small. In addition, the strength of the specimens after carbonation curing showed a tendency to decrease rather than increase as the carbonation curing starting point progressed. Thus, the most favorable carbonation curing starting point is 4 h after the start of hydration, with the best results of early-age carbonation curing and gradually decreasing results of later carbonation curing.

**Figure 11:**
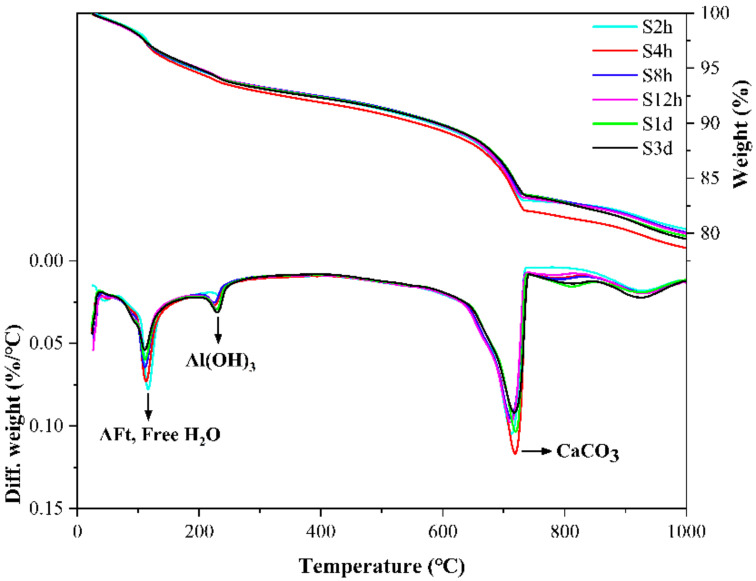
TG-DTG curves of CSA cement pastes corresponding to different carbonation starting points.

Figure 11 shows the TG-DTG curves of samples at different carbonation starting points, where the letter “S” indicates the starting point of the early-age carbonation curing. In the temperature range of 80–150 °C, which corresponds to the thermal decomposition of AFt, the earlier the carbonation starting point, the higher the mass loss peak of AFt, indicating that the amount of AFt is higher, which indicates that the occurrence of the carbonization reaction can promote the hydration of the cement paste. In the temperature range of 600–800 °C, the mass loss peak corresponding to S4h is the highest, followed by S2h, and the mass loss peak of S3d is the lowest. This phenomenon further illustrates that the most favorable time point for the paste carbonization reaction is 4 h after initial hydration, and the effect of early-age carbonation curing is much better than that of later carbonation curing. This is because as the hydration progresses, the paste becomes denser and the porosity becomes lower, so it is increasingly difficult for CO_2_ to diffuse into the paste. When carbonization is carried out at the early stage of hydration, the CO_2_ diffuses more easily into the paste and the hydration and carbonization reactions proceed simultaneously and mutually reinforce each other, thus greatly increasing the compressive strength of the specimen.

**Figure 12:**
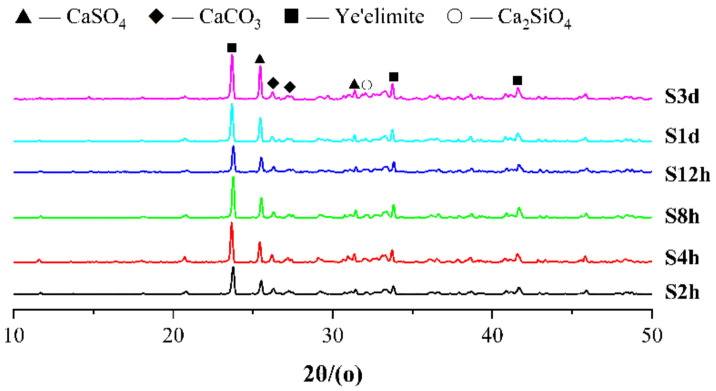
XRD patterns of CSA cement pastes corresponding to different carbonation starting points.

**Figure 13:**
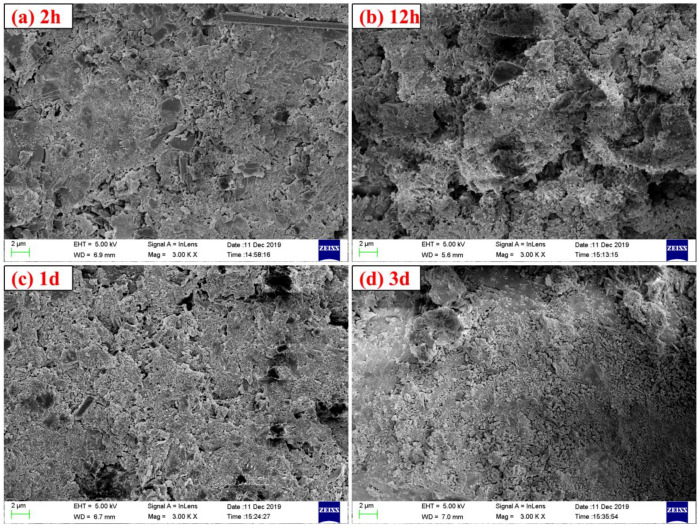
SEM images of CSA cement pastes corresponding to different carbonation starting points: (**a**) 2 h, (**b**) 12 h, (**c**) 1 d, (**d**) 3 d.

Figure 12 shows the XRD patterns of the specimens with different carbonation starting time points, and it can be seen that the earlier the carbonation starting point is, the lower the diffraction peak intensity of ye’elimite and calcium sulfate is, indicating that the earlier the carbonation starting point is, the more favorable it is for the hydration carbonization reaction of minerals in the CSA cement paste. Like the test results of w/b ratio, there is no obvious diffraction peak of AFt and CaCO_3_ in the XRD pattern, indicating that AFt has reacted under the action of carbonation curing, and the crystal structure has changed. The crystal structure of CaCO_3_ formed by carbonization is irregular, so it cannot be detected by XRD. Figure 13 shows the SEM images of the samples with different carbonation starting points. It can be seen that the differences observed under the scanning electron microscope (SEM) for the samples with different carbonation starting points are not very obvious. This is related to the local difference of the cement paste. It also shows that different carbonation starting points will not change the types of carbonization reaction and hydration reaction products, but will affect the degree of reaction and macroscopic performance. In Figure 13, there is no large number of needle-shaped AFt or regular cube-shaped CaCO_3_, which is consistent with the XRD results.

### 3.4. Carbonation Curing Pressure

**Figure 14:**
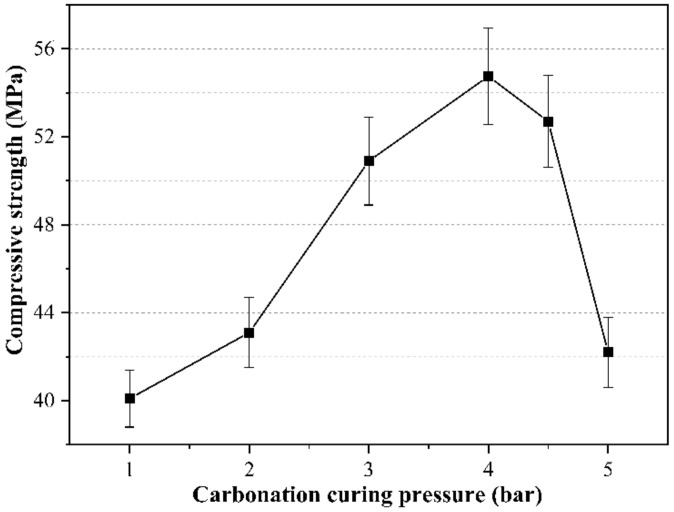
Influence of carbonation curing pressure on the compressive strength of CSA cement pastes.

In the test to study the influence of curing pressure on the properties of CSA cement paste, the water–binder ratio is 0.15, the briquetting pressure is 20 MPa, and the carbonation time point is 4 h. The change in the properties of CSA cement paste after 2 h of carbonization curing with six groups of different carburizing curing pressure is studied. Figure 14 shows the influence of different carbonation curing pressures on the compressive strength of the paste. When the curing pressure does not exceed 4 bar, the compressive strength of the paste gradually increases as the curing pressure increases.

The compressive strength is inversely proportional to the curing pressure when the curing pressure exceeds 4 bar. The maximum compressive strength is 54.8 MPa at a curing pressure of 4 bar. This shows that an appropriate increase in curing pressure is conducive to the occurrence of the paste carbonization reaction, which can enhance the early properties of the paste. If the curing pressure is too high, the paste structure will be destroyed, resulting in a great decrease in strength.

**Figure 15:**
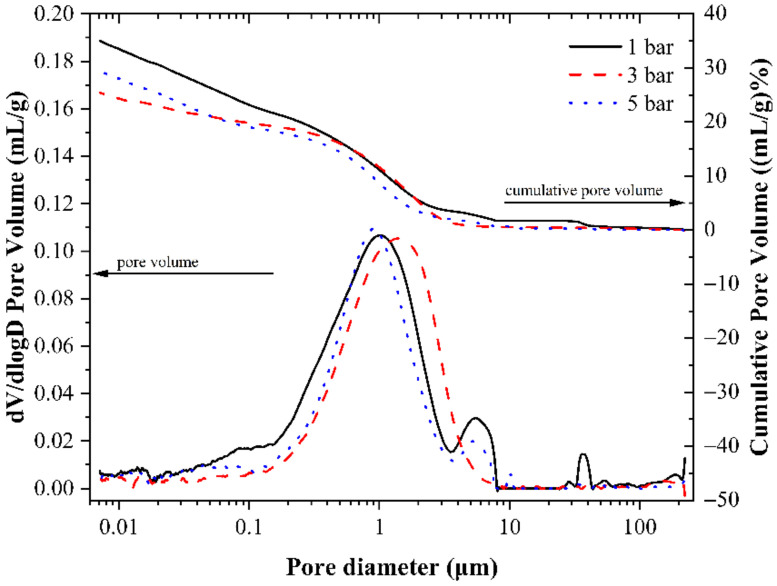
Pore volume distribution of CSA cement pastes corresponding to different carbonation curing pressures.

The cumulative porosity curve and porosity differential curve of the sample are shown in Figure 15. The sample with a curing pressure of 3 bar has the lowest cumulative porosity, followed by the sample with a curing pressure of 5 bar, and the sample with a curing pressure of 1 bar has the highest cumulative porosity. Increasing the curing pressure is beneficial to the carbonization reaction of the paste, and the occurrence of the carbonization reaction is conducive to refining the pores of the paste and making the pore distribution of the paste more uniform. Therefore, the cumulative porosity of the sample under the curing pressure of 3 bar is lower than that of the sample under the curing pressure of 1 bar. Although the sample with a curing pressure of 5 bar can undergo a large amount of carbonization reaction, the microstructure inside the paste is also destroyed under higher gas pressure, thereby increasing the porosity. According to the cumulative porosity differential curves in Figure 15, the differential curve of the specimen with a curing pressure of 3 bar fits the normal distribution more perfectly than those of the other two groups of specimens, indicating a more uniform pore distribution and more concentrated pore diameter in the specimen with a curing pressure of 3 bar. The pore diameters corresponding to the highest peaks of the differential curves of the samples with curing pressures of 1 bar and 5 bar are smaller, indicating that the most distributed pore diameters in these two groups of samples are smaller than those with a curing pressure of 3 bar. However, these two groups of samples also have many pores with a pore diameter of more than 5 μm, and the pore distribution is not uniform, so the compressive strength is lower than that of the sample with a curing pressure of 3 bar. Therefore, the most suitable curing pressure for early-age carbonation curing is between 3 and 4 bar.

**Figure 16:**
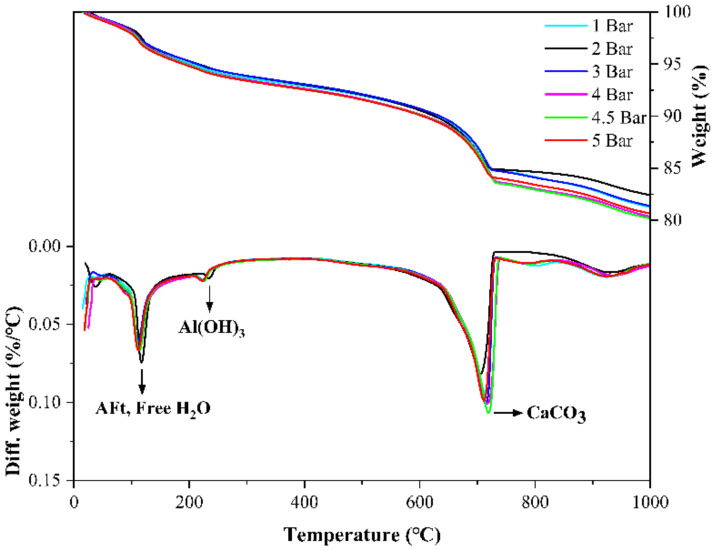
TG-DTG curves of CSA cement pastes corresponding to different carbonation curing pressures.

**Figure 17:**
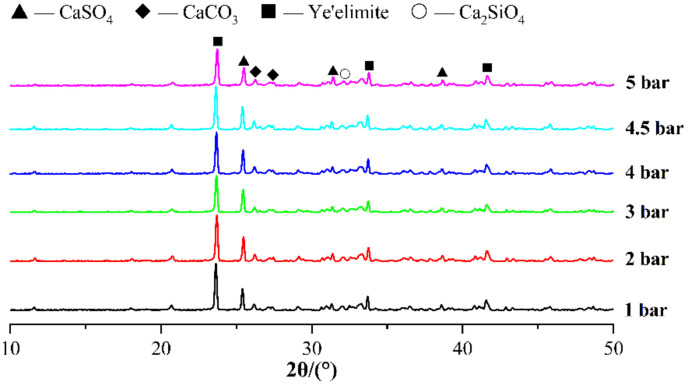
XRD patterns of CSA cement pastes corresponding to different carbonation curing pressures.

**Figure 18:**
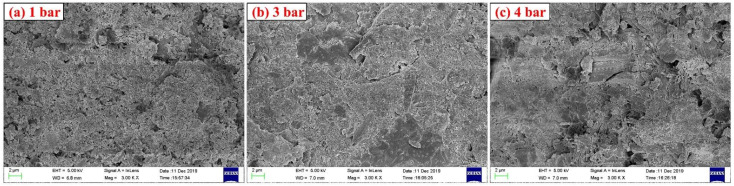
SEM images of CSA cement pastes corresponding to different carbonation curing pressures: (**a**) 1 bar, (**b**) 3 bar, and (**c**) 4 bar.

Figure 16 shows the TG-DTG curves for specimens with different carbonation curing pressures, and the weight loss peaks of specimens with high carbonation curing pressures are significantly higher than those with low curing pressures in the temperature ranges of 80–150 °C and 600–800 °C. In the XRD pattern in Figure 17, the higher the carbonation curing pressure is, the lower the diffraction peaks of ye’elimite and calcium sulfate are. From the results of TG and XRD, the increase in carbonation curing pressure can always promote the hydration process of CSA cement paste, so that cement minerals will be hydrated, carbonized, and consumed faster. In addition to affecting the degree of carbonation and hydration, the curing pressure will also affect the diameter and distribution of the microscopic pore structure of the paste, resulting in changes in the macroscopic mechanical properties. As with the previous test results, there are no obvious AFt and CaCO_3_ diffraction peaks in the XRD pattern. Figure 18 shows the SEM images of samples with different curing pressures, and the comparison shows that an increase in carbonation pressure results in a denser microstructure of the paste and more hydrated and carbonized products, which is consistent with the results of other experiments.

### 3.5. Carbonation Curing Time

**Figure 19:**
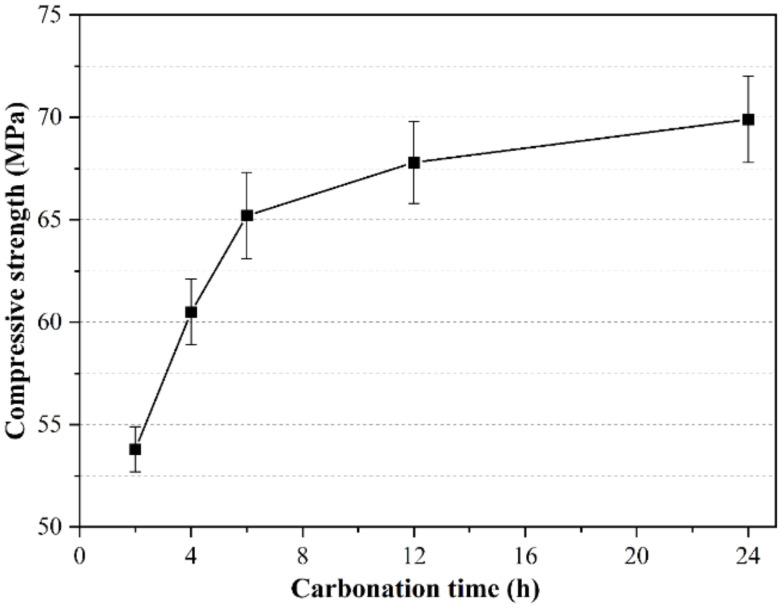
Influence of carbonation curing time on compressive strength of CSA cement pastes.

As with the previous test, this section still uses a water–binder ratio of 0.15. Keeping the briquetting pressure of 20 MPa unchanged, the carbonation starting point is 4 h after the start of hydration, and the carbonation curing pressure is 4 bar. In order to ensure that the hydration time of the paste is the same, this test uses different carbonation curing times to cure the samples, and they are all put into the curing box for standard curing to 1 d before testing to avoid the difference in hydration time caused by the test result error. Figure 19 shows the compressive strength test results of CSA cement paste under different carbonation curing times. In the early stage of CSA cement paste hydration, the compressive strength increases with the increase in carbonation curing time. This is because the longer the carbonation time is, the higher the degree of carbonization of the paste is, so the higher the early strength of the paste is. When the carbonation curing time is 24 h, the compressive strength is as high as 70 MPa, which is higher than the 28 d standard curing strength of the paste. When the carbonation curing time was gradually increased, the increase in the compressive strength of the specimen decreased gradually, indicating that the effect of carbonation curing time on the strength of the specimen tends to be steady. This is because increasing the curing time will cause more and more mineral phases and hydration product phases to react with CO_2_ in the paste. After all the carbonizable substances in the paste have reacted, continuing to extend the carbonation time will have no effect on the increase in strength. When the carbonation curing time is 8 h, the compressive strength increases the most.

**Figure 20:**
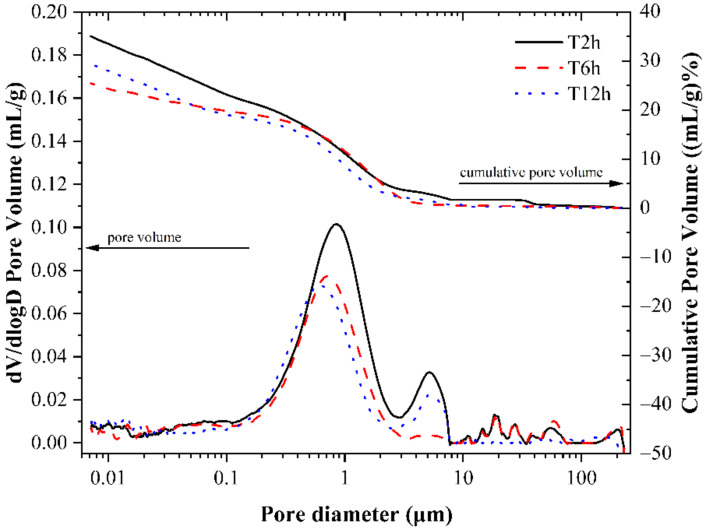
Pore volume distribution of CSA cement pastes corresponding to different carbonation curing times.

Figure 20 shows the cumulative porosity curves and corresponding differential curves for specimens with different carbonation curing times. The letter “T” indicates the total time of the early-age carbonation curing. From Figure 20, the cumulative porosity of the specimen gradually decreases as the carbonation curing time increases, indicating that increasing the carbonation curing time can improve the pore structure of the paste and reduce the porosity of the specimen. The pore structure distribution in the paste tends to conform to a normal distribution, and the pore diameter corresponding to the highest peak of the cumulative porosity differential curve of the three groups of specimens is inversely proportional to the carbonation curing time, i.e., the longer the carbonation curing time, the smaller the pore diameter of the pore with the highest percentage of pores in the paste. For the T12h sample, there were almost no pores larger than 7.74 μm, while pores with a diameter of 20 μm or even larger were also present in the T2h and T6h samples. This further illustrates that the prolongation of the carbonation time can improve the pore structure and the uniformity of the pore distribution of the paste, thus improving the macro-mechanical properties of the paste.

**Figure 21:**
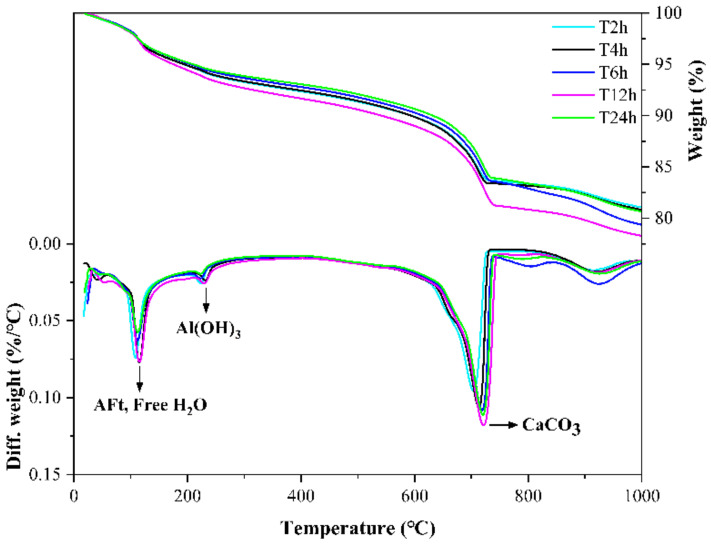
TG-DTG curves of CSA cement pastes corresponding to different carbonation curing times.

**Figure 22:**
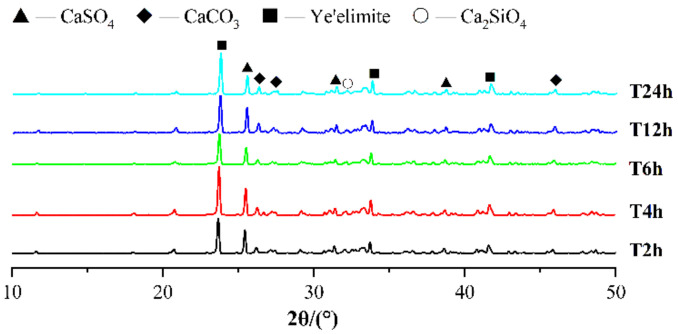
XRD patterns of CSA cement pastes corresponding to different carbonation curing times.

**Figure 23:**
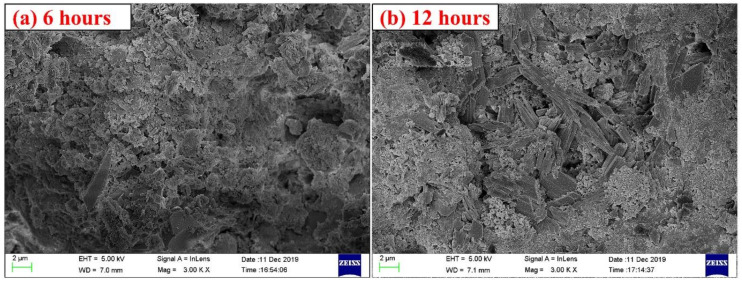
SEM images of CSA cement pastes corresponding to different carbonation curing times: (**a**) 6 h, (**b**) 12 h.

Figure 21 and Figure 22 show the TG-DTG curves and XRD patterns of CSA cement pastes with different carbonation curing times. From Figure 21, in the temperature range of 80–150 °C, the weight loss peak of T12h is the highest, followed by T6h, and the weight loss peak of T24h is almost the lowest, which shows that the mutual promotion of carbonization reaction and hydration reaction does not always exist, and blindly extending the carbonation time cannot make the paste more hydrated, because both carbonization reaction and hydration reaction need the participation of water, and when the carbonation curing time is too long, the paste will lack the water involved in the reaction, thus affecting the reaction. In the temperature range of 600–800 °C, the weight loss peak of T12h is the highest, followed by T24h and T6h, and the weight loss peak of T2h is the lowest, indicating that the proper extension of carbonation time is beneficial to the full carbonization reaction of the mineral phase and hydration product phase inside the paste. However, the carbonation curing time is too long, which limits the hydration reaction, which is not conducive to the progress of carbonization. From the XRD patterns in Figure 22, the diffraction peaks of ye’elimite and calcium sulfate in the T6h sample are the lowest, indicating that the cement mineral phase in the T6h sample has the most complete reaction. The diffraction peaks of AFt and carbonate are also not evident on the XRD pattern. From this, it can be concluded that the most suitable carbonation curing time is 6 to 12 h, which is beneficial for both carbonation and hydration reactions. Figure 23 shows the SEM images of the T6h and T12h samples. The substances in the T6h sample are more closely packed, and the reaction products in the T12h sample are more loosely packed, with more granular substances, which shows that the T6h sample is more hydrated and the T12h specimen has a higher degree of carbonization reaction.

## 4. Conclusions

This research advances our understanding of the early-age carbonation of calcium sulfoaluminate (CSA) cement-based materials. The current results of carbonation experiments conducted by MIP, TG-DTG, XRD, and SEM lead to the following conclusions.

1. Early-age carbonation curing can effectively promote the hardening speed of CSA cement, greatly improving the early strength.
2. Early-age carbonation curing can reduce the cumulative porosity of the CSA cement paste and refine the pore diameter distribution, resulting in a more uniform pore diameter distribution.
3. The optimized performance mainly depends on two aspects of physics and chemistry. The first is the physical pressurization of carbonation curing; more importantly, it accelerates the carbonization reaction to produce amorphous calcium carbonate (CaCO_3_) and aluminum hydroxide (AH_3_) gel, which makes the microstructure of the CSA cement paste more compact.
4. The technical parameters for early-age carbonation curing of CSA cement are recommended as follows: a w/b ratio of 0.15–0.22, carbonization curing for 6–12 h after 4 h initial hydration, with carbonization curing pressure at 3–4 bar.

## Figures and Tables

**Table 1:** Chemical composition of CSA cement (wt.%) [26].

SiO_2_	Al_2_O_3_	Fe_2_O_3_	CaO	MgO	K_2_O	Na_2_O	SO_3_	TiO_2_	CO_2_	Total
6.03	18.77	2.44	47.56	1.75	0.77	0.08	16.22	0.9	5.11	99.63

**Table 2:** Physical properties of cement.

Test Content	SSA (m^2^/kg)	Setting Time (min)		Flexural Strength (MPa)			Compressive Strength (MPa)		
		Initial	Final	6 h	1 Day	3 Days	6 h	1 Day	3 Days
Standard requirement	≥350	≥12	≤180	4.0	5.5	6.0	20.0	35.0	42.5
Measured value	446	22	38	6.7	7.4	8.5	30.1	44.5	50.7

SSA = specific surface area.

## Data Availability

The data underlying this article will be shared on reasonable request from the corresponding author.
